# Nuclear lipid droplets: a novel regulator of nuclear homeostasis and ageing

**DOI:** 10.18632/aging.206175

**Published:** 2024-12-09

**Authors:** Konstantinos Palikaras, Nektarios Tavernarakis

**Affiliations:** 1 Department of Physiology, Medical School, National and Kapodistrian University of Athens, Athens, Greece; 2 Institute of Molecular Biology and Biotechnology, Foundation for Research and Technology - Hellas, Crete, Greece; 3 School of Medicine, University of Crete, Heraklion, Crete, Greece

**Keywords:** aging, ATGL-1, HLH-30/TFEB, lipid droplet, non-linear optical phenomena, nucleus

## Abstract

Aging is a fundamental driver of numerous life-threatening diseases, significantly compromising cellular structures and functions, including the integrity of the nucleus. A consistent feature of aging across diverse species is the progressive accumulation of lipid droplets (nLDs) within the nuclear compartment, which disrupts nuclear architecture and functionality. Notably, aging is accompanied by a marked increase in nLD accumulation at the nuclear envelope. Interventions known to extend lifespan, such as caloric restriction and reduced insulin signaling, significantly reduce both the rate of accumulation and the size of nLDs. The triglyceride lipase ATGL-1, which localizes to the nuclear envelope, plays a critical role in limiting nLD buildup and maintaining nuclear lipid balance, especially in long-lived mutant worms. These findings establish excessive nuclear lipid deposition as a key hallmark of aging, with profound implications for nuclear processes such as chromatin organization, DNA repair, and gene regulation. In addition, ATGL-1 emerges as a promising therapeutic target for preserving nuclear health, extending organismal healthspan, and combating age-related disorders driven by lipid dysregulation.

Ageing is a multifaceted biological process that manifests as gradual decline of cellular functions, and ultimately leads to deterioration of organismal physiology. This complex phenomenon is influenced by a variety of factors, including genetic, environmental, and metabolic effects [1, 2]. A prominent hallmark of ageing is the accumulation of dysfunctional cellular components, which disrupts tissue homeostasis, contributing to age-related pathologies [3, 4]. This feature includes the abnormal deposition of lipids in non-adipose tissues, known as ectopic fat, which is highly associated with metabolic syndrome and various age-related conditions, including cardiovascular disease, type 2 diabetes, and neurodegenerative disorders, among others, [5, 6].

Lipid droplets (LDs) are primarily recognized as cytoplasmic organelles and have a pivotal role in energy storage, lipid metabolism, and cellular homeostasis. Traditionally, LDs presence has been confined to the cytoplasm, where they serve as reservoirs of neutral lipids, supporting energy balance and membrane biosynthesis [7, 8]. For a long time, the association between LDs and the nucleus was under-investigated. However, recent studies have unveiled an unexpected aspect of lipid biology demonstrating that LDs can also exist within the nucleus, forming nuclear lipid droplets (nLDs) [9–15]. Although LDs are typically generated from ER membranes and remain closely associated with the outer nuclear membrane, recent studies provide compelling evidence of LDs residing within nucleus [7, 8, 16, 17]. This nuclear localization is surprising and points to previously unappreciated roles for lipid storage and metabolism within the nucleus, introducing an additional layer of complexity to our understanding of cellular processes, particularly in the context of ageing.

Over the last decade, several methods have been developed to monitor LDs number, morphology, and distribution within cells and across tissues. However, many of these methods face limitations that prevent the real-time, *in vivo* tracking of LDs throughout the lifespan of an organism [18–21]. In previous studies, we have established the use of non-linear imaging modalities to assess LDs formation and deposition during ageing *in vivo* [21–23]. Using this label free and non-invasive methodology, we revealed that LDs progressively accumulate in the nuclear envelope as *C. elegans* ages [24]. Recent findings suggest that the nuclear envelope is a metabolically active site where lipids are synthesized and stored, adding a new dimension to our understanding of lipid metabolism and its relationship with nuclear integrity [12, 15]. Notably, the build-up of nuclear lipid droplets (nLDs) is closely associated with age-related alterations in the nuclear lamina protein LMN-1, the *C. elegans* homolog of mammalian lamin A/C. These findings underscore an intricate relationship between nuclear envelope components and LDs functionality. These interactions could be crucial for the maintenance of nuclear integrity, as changes in nuclear morphology are well-established hallmarks of ageing across species [25, 26]. Further supporting this notion, studies in human cells have shown that mutations in the *LMNA* gene not only lead to abnormal nuclear morphology but also result in altered expression of metabolism-related genes, features similarly observed in cells derived from metabolic syndrome patients, which also exhibit impaired nuclear distribution of lamin A/C [27, 28]. The accumulation of nLDs, therefore, is not simply a passive consequence of ageing but might actively contribute to nuclear morphology disruption and impaired cellular function. Notably, disruptions in nuclear lipid homeostasis have been linked to metabolic disorders such as fatty liver disease and obesity, suggesting that similar mechanisms could be conserved across species and relevant not only to ageing but also to disease pathology [5, 8, 25]. However, further investigation is needed to assess nLDs distribution within nuclear compartments particularly in cells derived from progeria and metabolic syndrome patients, where such mechanisms might have a critical role.

While the physical association of LDs with the nuclear compartments is highly recognized, our understanding of their functional roles in nuclear homeostasis remains elusive. Genetic studies conducted in mammalian cells, flies and nematodes have been shown that LDs contribute to nuclear integrity by storing histones, binding transcription factors, directly interacting with other proteins that regulate the transport between the cytoplasm and nucleoplasm [7, 8, 16]. Hence, the uncontrolled accumulation of LDs in the nuclear envelope might be detrimental for several nuclear processes, including nuclear transport, DNA repair, chromatin remodelling and ultimately, gene expression. A recent study in *C. elegans* revealed that nLDs are coated by LMN-1 and/or heterochromatin, indicating that the accumulation of nLDs could play a role in the disposal of peripheral heterochromatin [14]. These results indicate that nLDs might influence chromatin organization, given their formation at the nuclear envelope and their ability to penetrate the nuclear lamina, entering the nucleoplasm. This is particularly relevant since the loss of heterochromatin and the derepression of silenced genes are considered major contributors to premature ageing. Chromatin loosening is a common feature of ageing, leading to deregulated gene expression and compromised genome integrity [29, 30]. Lamin-Associated Domains (LADs), which are closely linked to heterochromatin, could be destabilized by the age-dependent accrual of nLDs at the nuclear lamina (Figure 1). In turn, these events could contribute to heterochromatin loss during ageing, suggesting that nLD accumulation may play a significant role in age-related chromatin remodelling. Moreover, the excessive nLD accumulation could interfere with chromosome territories, leading to nuclear damage and cellular dysfunction. These concepts are further supported by the well-documented loss of intestinal nuclei during *C. elegans* ageing, which might, in fact, be driven by increased nLD accumulation [14, 31, 32]. However, further studies are needed to validate these concepts and uncover the precise mechanisms involved.

**Figure 1:**
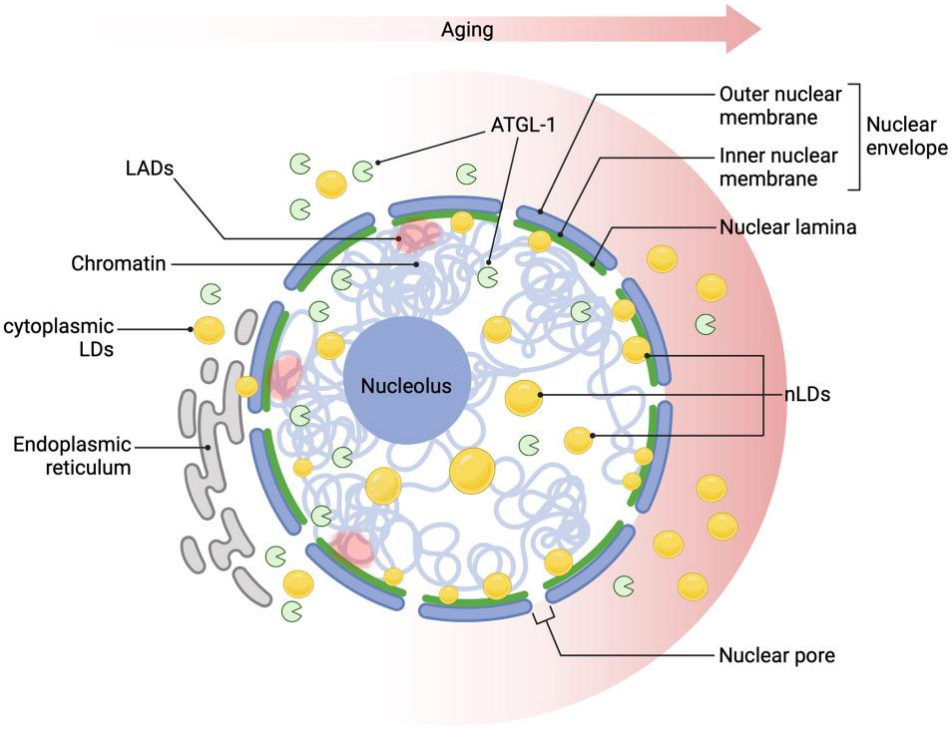
**Age-dependent nLDs accumulation affects nuclear homeostasis.** Over time, lipid droplets (LDs) accumulate progressively in the cytoplasm and within the nuclear lamina, closely associated with age-related changes in the nuclear lamina protein LMN-1 (the nematode homolog of lamin A/C), which supports nuclear morphology. This nLD accumulation may disrupt Lamin-Associated Domains (LADs), chromatin regions anchored to the nuclear periphery, leading to chromatin remodelling, heterochromatin destabilization, and compromised nuclear integrity, which are well-established hallmarks of ageing. Longevity-promoting interventions activate the lipase ATGL-1, regulated by HLH-30/TFEB, linking lipid metabolism to pathways that promote cellular longevity. These changes in nLDs could impact key nuclear processes, including gene expression and DNA repair, thereby contributing to cellular ageing. Created in BioRender (Palikaras, K., 2024, https://BioRender.com/t49f941).

One of the most compelling aspects of our study is the effect of longevity-promoting interventions on nLD accumulation. Dietary restriction and reduced insulin signalling were found to significantly reduce both the size and number of nLDs in aged nematodes. These findings underscore the role of metabolic regulation in extending lifespan, hinting at the importance of nLDs homeostasis in maintaining cellular integrity during ageing. Both longevity-promoting interventions correlated with lower levels of nLD accumulation, preservation of nuclear morphology, and sustained expression of the nuclear structural protein LMN-1 [24, 33, 34]. Furthermore, our study uncovered the transcription factor HLH-30, the nematode homolog of the mammalian transcription factor EB (TFEB), to play an essential role in preserving nuclear lipid balance. Intriguingly, HLH-30 operates independently of autophagy, establishing a distinct pathway for nLDs regulation. Both HLH-30 and its mammalian counterpart TFEB are activated and translocate to the nucleus in response to low insulin signalling and caloric restriction [35, 36]. HLH-30 subsequently regulates the expression of genes involved in autophagy, lysosomal function, and lipid metabolism, including the gene for the lipase ATGL-1 [24, 35, 36]. Our findings also indicate that ATGL-1 is essential for regulating nLD size and abundance in long-lived nematodes, as its overexpression alone is sufficient to maintain nLDs. Notably, ATGL-1 is localized in both the cytoplasm and nuclear envelope, suggesting it plays a dual role in moderating lipid accumulation across cellular compartments [24]. The mammalian homolog of ATGL-1, PNPLA2, exhibits similar dual localization, being found in both cytosolic LDs and the nucleoplasm in a range of human cell lines, including A-549, U-251MG, HEK293, U2OS, and SiHA cells (as documented in the Human Protein Atlas, http://www.proteinatlas.org/). The regulatory relationship between HLH-30 and ATGL-1 provides a mechanistic link between longevity-promoting pathways and nLDs metabolism, offering insights into how these interventions maintain nuclear integrity through lipid homeostasis. Taken together, these findings suggest that metabolic pathways supporting longevity may promote cellular health by directly influencing nLD dynamics. This influence, in turn, likely plays a critical role in sustaining nuclear integrity, positioning nLD regulation as a promising target for interventions aimed at promoting healthy ageing.

The identification of age-dependent nLDs accumulation as a hallmark of ageing marks a significant advancement in our understanding of the interplay between lipid metabolism and nuclear function. Accumulation of nLDs disrupts nuclear integrity, potentially impairing essential nuclear processes such as chromatin organization, DNA repair, and gene expression. Beyond advancing our knowledge of the ageing process, this discovery suggests promising targets for interventions aimed at extending healthspan and preventing age-related diseases. As research into nLDs regulation progresses, it is conceivable that novel therapeutic strategies will emerge, leveraging lipid metabolism to combat the effects of ageing and enhance human health. Given the established links between lipid deregulation, metabolic disorders, and ageing, these findings hold considerable promise for developing therapies aimed at safeguarding nuclear function and mitigating the adverse effects of ageing on cellular and organismal physiology.
